# Zebrafish tissue injury causes upregulation of interleukin-1 and caspase-dependent amplification of the inflammatory response

**DOI:** 10.1242/dmm.013029

**Published:** 2013-11-07

**Authors:** Nikolay V. Ogryzko, Emily E. Hoggett, Sara Solaymani-Kohal, Simon Tazzyman, Timothy J. A. Chico, Stephen A. Renshaw, Heather L. Wilson

**Affiliations:** 1 Medical Research Council Centre for Developmental and Biomedical Genetics, University of Sheffield, Sheffield, S10 2TN, UK.; 2 Department of Cardiovascular Science, University of Sheffield, Sheffield, S10 2RX, UK.; 3 Academic Unit of Inflammation and Tumour Targeting, Department of Oncology, University of Sheffield, Sheffield, S10 2RX, UK.; 4 Department of Infection and Immunity and MRC Centre for Developmental and Biomedical Genetics, University of Sheffield, Sheffield, S10 2RX, UK.

**Keywords:** Inflammation, Interleukin-1, Zebrafish

## Abstract

Interleukin-1 (IL-1), the ‘gatekeeper’ of inflammation, is the apical cytokine in a signalling cascade that drives the early response to injury or infection. Expression, processing and secretion of IL-1 are tightly controlled, and dysregulated IL-1 signalling has been implicated in a number of pathologies ranging from atherosclerosis to complications of infection. Our understanding of these processes comes from *in vitro* monocytic cell culture models as lines or primary isolates, in which a range and spectra of IL-1 secretion mechanisms have been described. We therefore investigated whether zebrafish embryos provide a suitable *in vivo* model for studying IL-1-mediated inflammation. Structurally, zebrafish IL-1β shares a β-sheet-rich trefoil structure with its human counterpart. Functionally, leukocyte expression of IL-1β was detectable only following injury, which activated leukocytes throughout zebrafish embryos. Migration of macrophages and neutrophils was attenuated by inhibitors of either caspase-1 or P2X7, which similarly inhibited the activation of NF-κB at the site of injury. Zebrafish offer a new and versatile model to study the IL-1β pathway in inflammatory disease and should offer unique insights into IL-1 biology *in vivo*.

## INTRODUCTION

Interleukin-1 (IL-1) is an important activator of inflammation. Dysregulated IL-1β function has been described in the pathology of a number of auto- or chronic inflammatory diseases, leading to this cytokine being described as the ‘gatekeeper’ of inflammation (Dinarello, 2011c). Despite its key role in initiating inflammation, many aspects of IL-1β activity remain poorly understood, in part due to its unconventional secretion mechanism but also due to the complex array of proteins involved in its activation (Schroder and Tschopp, 2010). In macrophages, *IL-1β* transcription is induced and *IL-1β* mRNA stabilised following detection of pathogen-derived Toll-like receptor (TLR) ligands (Bufler et al., 2004). Processing and secretion of inactive pro-IL-1β requires a secondary stimulus, the best-studied being ATP, a key damage-associated molecular pattern (DAMP) (Lister et al., 2007). ATP activates the P2X7 receptor, resulting in rapid assembly of the inflammasome, an IL-1β-activation and -processing platform. IL-1β is thereby processed into its active form, with concomitant secretion (Rathinam et al., 2012).

IL-1β secretion is proposed to occur via a number of different mechanisms, ranging from lysosomal and microvesicular to pyroptotic, dependent on the strength of the inflammatory stimulus and the cell type in question (López-Castejón and Brough, 2011). Our understanding of these mechanisms is built predominantly on cell-culture studies of various cell types, although, additionally, animal models have been used to evaluate the requirement of specific proteins in IL-1β-mediated inflammation (Horai et al., 2000; Kuida et al., 1995). However, it has not been possible to combine the key features of such models to determine, in an intact organism, the vesicular component of IL-1β secretion and how IL-1β is specifically targeted to effector cells.

The evolutionary origins of innate immunity predate the first vertebrates, with cytokine signalling pathways detected in simple organisms (Beck and Habicht, 1991). Much of the complexity of the human immune system is well established in ray-finned fish, making zebrafish a tractable model to study innate immunity and inflammation *in vivo* (Renshaw and Trede, 2012). Importantly, zebrafish possess orthologues of the known components of IL-1β signalling, including TLRs, NF-κB, IL-1 receptors I and II, and P2X7 (Huising et al., 2004; López-Castejón et al., 2007; Stein et al., 2007). Here, we use the zebrafish model, *Danio rerio*, to study IL-1β signalling, and demonstrate its value as a model of IL-1β biology through characterising the induction of IL-1β in response to injury and the attenuation of inflammatory signals through the use of inhibitors of the IL-1β pathway.

## RESULTS

### Zebrafish and human IL-1β are structurally conserved

Previous studies describing the use of the zebrafish as a tool for investigating IL-1β have highlighted the differences between zebrafish and human IL-1β; specifically, the lack of a conserved caspase-1 cleavage site on the zebrafish homologue (Bird et al., 2002). The two proteins share only 27% amino acid identity, but we observed greater identity (31%) in the C-terminal domain, representing the mature cytokine. We submitted the zebrafish IL-1β sequence to the Phyre structural prediction server (Kelley and Sternberg, 2009) generating a β-sheet-rich trefoil structure closely matching that of the mature human cytokine (supplementary material Fig. S1).

### IL-1β message is induced in leukocytes throughout the zebrafish in response to injury

**Fig. 1.**
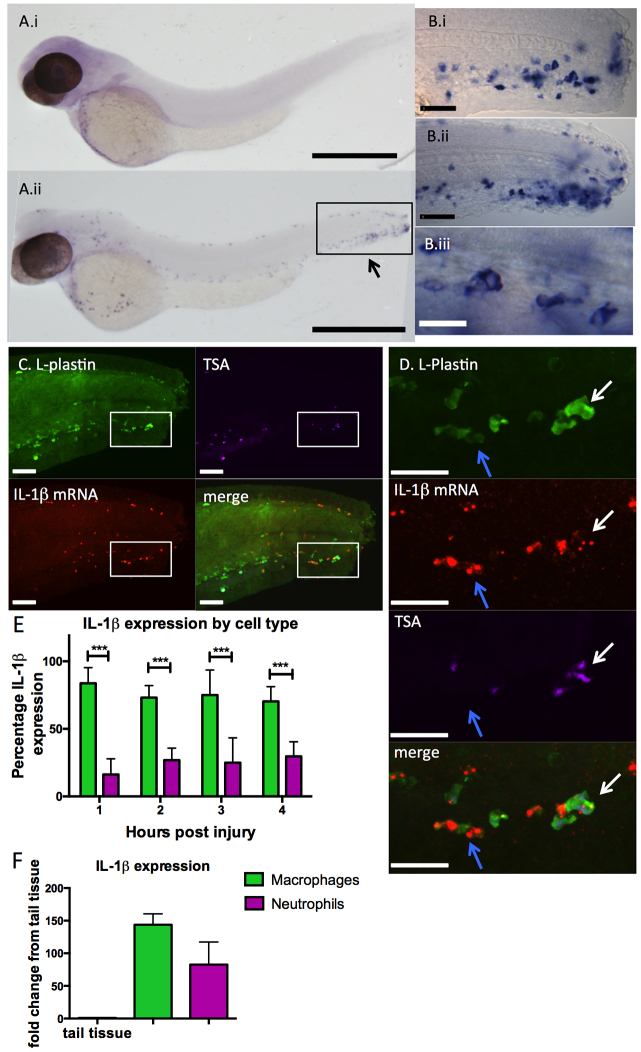
***IL-1β* expression is induced in leukocytes throughout the embryo in response to injury.** Expression analysis of *IL-1β* by *in situ* hybridisation. (Ai) Embryos fixed at 48 hours post-fertilisation (hpf) show no IL-1β expression before injury, but IL-1β expression can be detected in cells throughout the embryo 2 hours post injury (hpi) by tailfin transection (Aii). Arrow indicates area represented in Bi,ii. Scale bars: 500 μm. (Bi–ii) *IL-1β* expression at the site of injury appears localised to cells with typical leukocyte morphology: close up views of region represented in Aii by a box in (Bi) 24 hpf embryos at 2 hpi and (Bii) 48 hpf embryos 2 hpi. Scale bars: 50 μm. (Biii) Magnified image of tail region: *IL-1β*-positive cells have large nuclei and leukocytic morphology. Scale bar: 20 μm. (C) 48-hpf embryos were fixed and at 2 hpi were probed with anti-L-plastin (green; labelling leukocytes), stained for endogenous neutrophil peroxidase activity with Cy5 TSA (purple) and FISH performed to detect *IL-1β* mRNA (red) to determine the localisation of *IL-1β* in response to injury. Scale bars: 40 μm. (D) Close-up of boxed area shown in C. Scale bars: 40 μm. *IL-1β* was detected both in neutrophils (TSA^+^;L-plastin^+^, white arrows) and macrophages (TSA^−^;L-plastin^+^, blue arrows). (E) Quantification of *IL-1β*-expressing cells revealed expression predominantly in macrophages at all time points assayed (***P≤0.001 by multiple *t*-test with Bonferroni correction, *n*=8 performed as two independent experiments). (F) IL-1β was detected at very high levels by qRT-PCR in FACS-isolated zebrafish leukocytes when normalised to uninjured tail tissue.

Having determined structural conservation of zebrafish IL-1β to its mammalian orthologues, we studied its expression and regulation in developing zebrafish. We hypothesised that the tight control of IL-1β translation by regulation of message abundance is only removed in response to inflammatory stimuli, as in mammalian systems (Bufler et al., 2004). No *IL-1β* mRNA was detected in unstimulated embryos or larvae at 24 or 48 hours post-fertilisation (hpf) using whole-mount *in situ* hybridisation (WISH) (Fig. 1A); however, when we probed embryos fixed at various stages after injury, we observed high-intensity *IL-1β* mRNA staining (Fig. 1B) in cells with a mononuclear morphology (Fig. 1Bi–Biii) characteristic of macrophages. Recent evidence also supports the role of neutrophils in IL-1β signalling (Basran et al., 2013). Using the neutrophil-specific *mpx:EGFP* transgenic line, we sorted neutrophils and control cells with comparable scatter characteristics from 6-week-old zebrafish. Using microarray expression analysis, *IL-1β* mRNA was detectable in zebrafish neutrophils at a level 4.9-times higher than in control cells. Because IL-1β seemed to be expressed in both neutrophils and macrophages, we further characterised IL-1β-expressing cells by staining embryos for neutrophil and macrophage markers [as described previously (Feng et al., 2010; Prajsnar et al., 2012)] alongside fluorescent WISH for IL-1β. We detected IL-1β colocalising with both neutrophil and macrophage markers (Fig. 1C,D). The initial inflammatory response was characterised predominantly by IL-1β expression in macrophages, and there were fewer positively stained neutrophils (Fig. 1E). This pattern was observed until 5 hours post-injury (hpi), after which expression declined, supporting a role in zebrafish embryos for IL-1β in inflammation initiation, as is characteristic in human disease (Dinarello, 2011b). To further identify leukocyte-specific *IL-1β* mRNA expression, we FACS-sorted *mpeg:Gal4;UAS:Kaede*-labelled macrophages (Ellett et al., 2011) and *mpx:EGFP*-labelled neutrophils (Renshaw et al., 2006), and observed very high expression levels in both of these cell types by qRT-PCR (Fig. 1F).

**TRANSLATIONAL IMPACT**

**Clinical issue**

Interleukin-1 (IL-1) is the apical cytokine in a signalling cascade that drives the early inflammatory response to injury or infection. The expression, processing and secretion of IL-1 is normally tightly regulated and dysregulation of IL-1 is thought to underlie the chronic progressive inflammation that results in atherosclerosis (leading to stroke and myocardial infarction), type 2 diabetes and osteoarthritis. Based on *in vitro* experiments, IL-1 is thought to be released into the circulation and into tissues via several unusual and unconventional secretory mechanism(s). *In vitro*, the mechanism of IL-1 secretion that is used is dependent on cell type and on the strength of the inflammatory stimulus. However, it is not known which secretion mechanism occurs physiologically because IL-1 signalling has not been imaged *in vivo*.

**Results**

The evolutionary origins of human innate immunity predate the first vertebrates and the immune system of ray-finned fish mirrors much of the complexity of the human immune system. In this study, therefore, the authors investigate whether zebrafish is a suitable *in vivo* model in which to study IL-1 secretion and signalling. To this end, they show that the predicted structure of the zebrafish IL-1β protein shares significant similarities with the structure of human IL-1β. They report that *IL-1β* mRNA levels are increased in zebrafish in response to injury. Finally, they show that inhibitors of IL-1 processing prevent immune cell migration to the site of injury in zebrafish.

**Implications and future directions**

These findings support the functional conservation of IL-1β signalling between the mammalian and teleost lineages, and identify zebrafish as a new and versatile *in vivo* model in which to study the biology of IL-1, the ‘gate-keeper’ of inflammation. The role of the specific pathways and mechanisms that are required for IL-1 secretion and signalling in a live intact physiological system can now be investigated using this model species, which offers researchers the potential to identify anti-inflammatory targets and to assess the effectiveness of potential therapies for acute and chronic inflammation.

### IL-1β pathway inhibitors reduce leukocyte recruitment to the site of injury

**Fig. 2.**
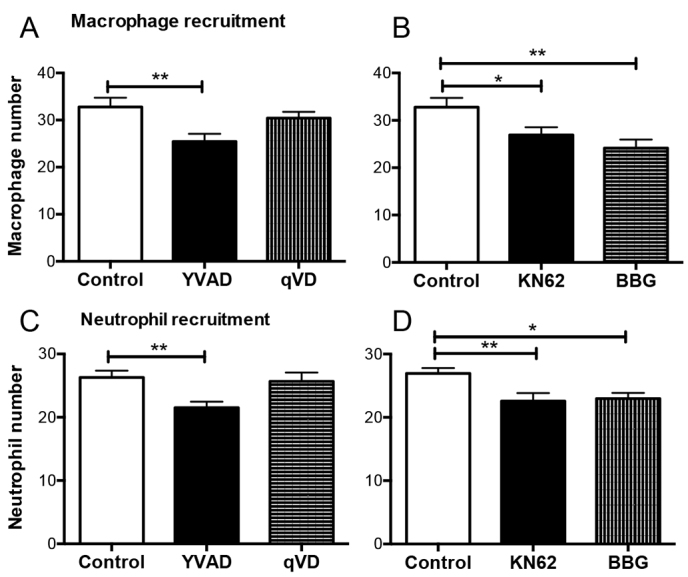
**Leukocyte recruitment to the site of injury is reduced by treatment with inhibitors of the IL-1β pathway.** (A,C) Zebrafish embryos treated with 50 μM YVAD showed reduced *mpeg1:gal4;UAS:Kaede*-labelled macrophage (A) and *mpx:EGFP*-labelled neutrophil (C) recruitment to the site of injury after 6 hours compared with DMSO-treated controls. qVD (50 μM) had no effect on leukocyte recruitment. (B,D) Both macrophage and neutrophil recruitment was also inhibited by treatment with P2X7 inhibitors KN62 (10 μM) and Brilliant Blue G (1 μM). *P≤0.05, **P≤0.01 by one-way ANOVA with Dunnett’s post-test, *n*=24 performed as three independent experiments.

The early enhancement of *IL-1β* mRNA in inflammatory leukocytes suggests a role in initiating the inflammatory response in the zebrafish tailfin injury model. By analogy to mammalian IL-1β processing and release, we tested the involvement of the inflammasome in IL-1β processing by using inhibitors. The recruitment of innate immune cells is a key feature of the inflammatory response. We therefore developed an assay based on macrophage and neutrophil recruitment following tissue injury. Using an *mpeg1:Gal4;UAS:Kaede* reporter line we measured a reduction in the number of macrophages recruited to the site of injury after 6 hours when treated either with the caspase-1 inhibitor YVAD or with the P2X7 antagonists KN62 or BBG (Fig. 2A,B). The pan-caspase inhibitor qVD did not attenuate macrophage recruitment, suggesting perhaps that the effect on inflammasome inhibition might be balanced *in vivo* by suppression of normal apoptosis. Neutrophil recruitment was similarly attenuated by YVAD, Brilliant Blue G and KN62, whereas qVD had no significant effect upon this response (Fig. 2C,D). The effect of IL-1β and P2X7 on macrophage recruitment was confirmed by morpholino knockdown of IL-1β (Roca et al., 2008) and P2X7 (Chang et al., 2011) (supplementary material Fig. S2).

### The caspase-1 inhibitor YVAD reduces the activity of NF-κB at the site of injury

**Fig. 3.**
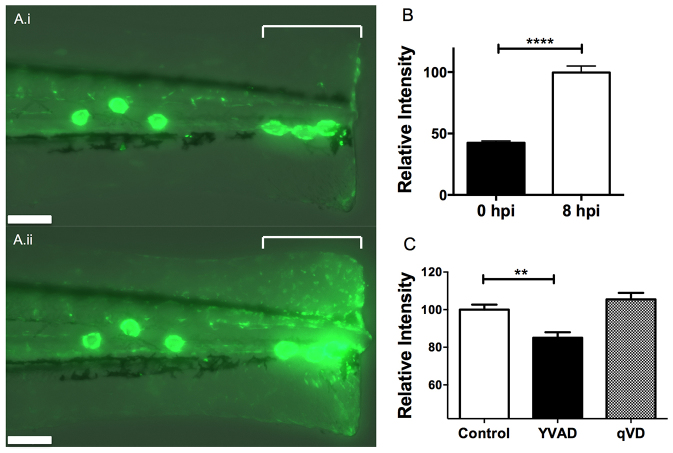
**Caspase-1 inhibitor YVAD downregulates NF-κB activation in response to injury.** (A) Fluorescence photomicrograph of *pNF-κB:EGFP* embryos following tailfin transection at 1 hpi (i) and 8 hpi (ii) indicating the region quantified (square bracket). Scale bars: 100 μm. (B) There is a 2.4-fold increase in EGFP fluorescence in response to injury, quantifiable as average fluorescent intensity across the transection site (indicated in A). ****P≤0.0001 by *t*-test. (C) Embryos treated with 50 μM YVAD show a reduction in EGFP fluorescence at 8 hpi compared with DMSO-treated controls and embryos treated with qVD. **P≤0.01 by one-way ANOVA with Dunnett’s post-test. *n*=30 performed as three independent experiments.

Because IL-1β acts on target cells via the IL-1 receptor activating NF-κB, we tested whether NF-κB signalling was sensitive to treatment with inflammasome inhibitors. We generated a transgenic line using the NF-κB reporter constructs previously described (Kanther et al., 2011) and confirmed that, in this line, NF-κB was activated in response to injury (Fig. 3A,B). Using this line, we developed an inflammation assay based on quantification of the mean fluorescent intensity at the site of tailfin transection and observed that, when treated with the caspase-1 inhibitor YVAD, the activation of NF-κB at the site of injury was significantly reduced (Fig. 3C); this effect was not seen with the pan-caspase inhibitor qVD. P2X7 antagonists showed a trend towards reduced NF-κB activation, although this effect was not statistically significant (data not shown).

### The expression of IL-1β in response to tailfin injury is reduced in response to treatment with IL-1β pathway inhibitors

**Fig. 4.**
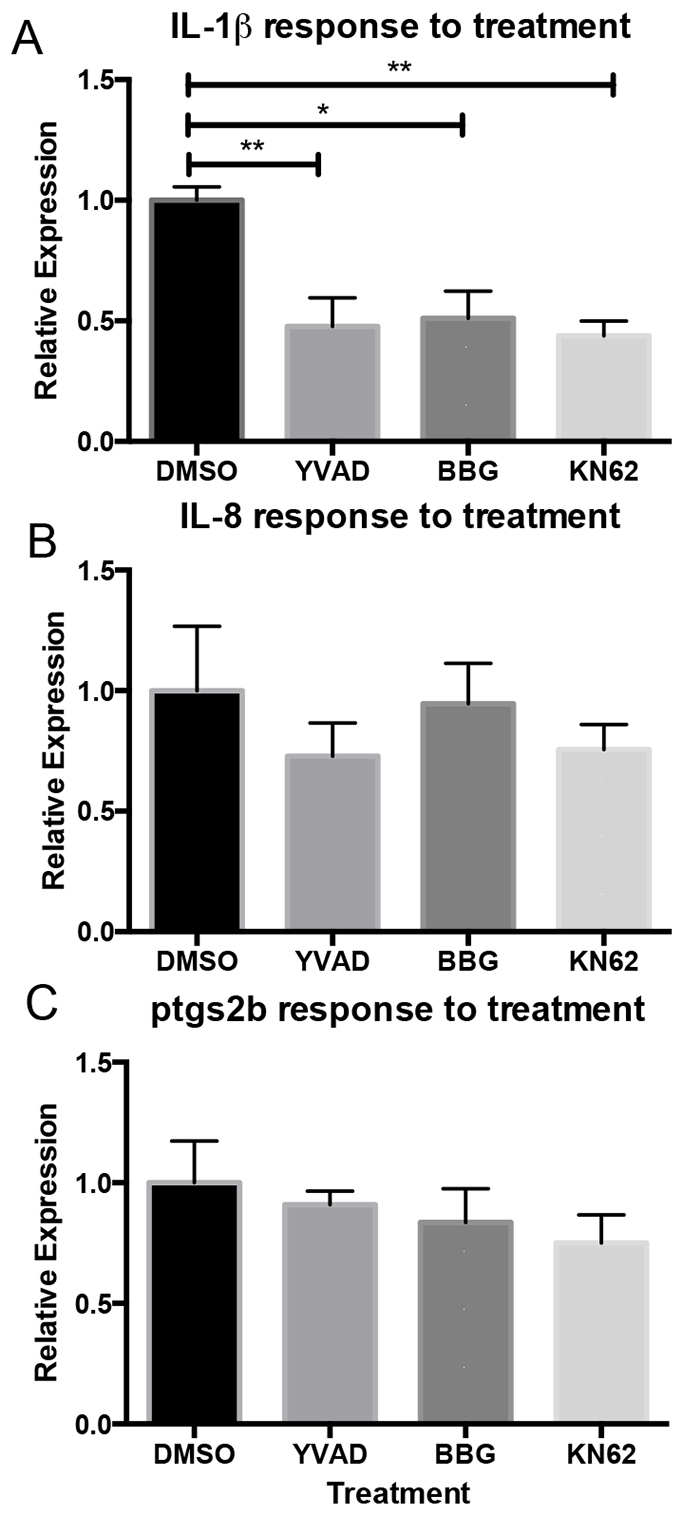
***IL-1β* expression in response to injury is reduced by treatment with YVAD, BBG and KN62.** Gene expression analysis shows a reduction in *IL-1β* expression in response to injury after treatment with YVAD, BBG or KN62, compared with other inflammatory markers (*IL-8a* and *ptgs2b*), which were unaffected. *P≤0.05 and **P≤0.01 by one-way ANOVA with Dunnett’s post-test of triplicate samples of at least three independent experiments.

Using the assay developed by de Oliveira et al. (de Oliveira et al., 2013), we investigated the expression of the same panel of inflammatory genes in response to tail injury and the effect of mammalian P2X7 and caspase-1 inhibitors BBG, KN62 and YVAD on expression of these genes. We tested whether zebrafish IL-1β induces its own downstream transcription, as described in numerous mammalian models (Dinarello, 2011a; Dinarello et al., 1987; Granowitz et al., 1992). We were able to detect the same induction of *IL-1β*, *IL-8a* and *ptgs2b* following injury as previously reported (de Oliveira et al., 2013) (supplementary material Fig. S3A–C). In addition, we measured a significant decrease in the expression of *IL-1β* but not *IL-8* or *ptgs2b* in response to treatment with YVAD, BBG or KN62 at 2 hpi (Fig. 4A). Taken together, these results demonstrate that the zebrafish inflammatory response is sensitive to inhibitors of inflammasome activity.

## DISCUSSION

The investigation of IL-1β pathway biology has hitherto relied primarily on *in vitro* cell-culture techniques. Observing these processes in a living model could yield valuable insights into the release mechanisms of this cytokine as the IL-1β-producing cells interact with the cellular microenvironment in an inflammatory lesion. These complex interactions are not accessible to conventional monoculture techniques. Zebrafish are an ideal model for *in vivo* observation of the vertebrate immune system (Henry et al., 2013), although investigation of IL-1β in zebrafish has been confounded by concerns relating to the conservation of IL-1β processing in the zebrafish (Angosto et al., 2012). Recent publications have described the cleavage of zebrafish IL-1β by the caspase-1 homologues caspase-A and caspase-B (Vojtech et al., 2012), suggesting conservation of IL-1β processing. The functional significance of caspase activity in inflammation in zebrafish and the implications for IL-1β function have not previously been explored. Here, we describe the temporal induction of the IL-1β message in response to injury and characterise this response in terms of the innate immune cell lineages responsible. Comparable to mammalian data (Morris et al., 2005), the primary IL-1β-expressing cell in the inflammatory response following tail transection is the macrophage, although neutrophils were also implicated.

The zebrafish innate immune response was reduced in magnitude following treatment with inhibitors of P2X7, a key activator of IL-1β processing in mammalian systems (Ward et al., 2010). These data are supported by the reduction in both cellular and transcriptional readouts of inflammation following treatment with the caspase-1 inhibitor YVAD, demonstrating a conserved role for this enzyme and for P2X7 in zebrafish immunity. Because apoptosis is widely regarded as an anti-inflammatory event (Fadok et al., 1992), it might be expected that inhibition of apoptosis with qVD leads to a reduction in apoptotic cells at the wound site, resulting in a net increase in the inflammatory burden at the site of injury, masking any underlying inhibition of IL-1β production. We also describe the reduction of *IL-1β* transcription following tissue injury by treatment with inhibitors of the IL-1 pathway, indicating that IL-1 amplifies the inflammatory response through further downstream IL-1 induction, similar to mammalian models. We were unable to detect the same reduction in expression of *IL-8* and *ptgs2b* in response to injury, suggesting that both IL-1-dependent and IL-1-independent pathways regulate proinflammatory gene transcriptions *in vivo*.

The structural conservation of IL-1β between humans and zebrafish, alongside the induction of IL-1β in leukocytes following injury and the ability of inhibitors of mammalian caspase-1 and P2X7 to attenuate inflammation support the functional conservation of IL-1β signalling between mammalian and teleost lineages. Here, we demonstrate the versatility and unique advantages of the zebrafish model in studying the IL-1β pathway *in vivo*. The use of transgenic zebrafish lines to directly visualise inflammation offers exciting future opportunities to observe IL-1β function *in vivo*.

## MATERIALS AND METHODS

### Transgenic line generation and maintenance

*Tg(mpeg1:Gal4.VP-16)sh256* and *Tg(pNF-κB:EGFP)sh235* were generated as previously described (Ellett et al., 2011; Kanther et al., 2011). *Tg(mpx:EGFP)i114* zebrafish were used to study neutrophils during inflammation (Renshaw et al., 2006). Zebrafish strains were maintained on a 14:10-hour light/dark cycle at 28°C as described (Nüsslein-Volhard and Dahm, 2002) in UK Home Office approved facilities in the MRC Centre for Developmental and Biomedical Genetics aquaria at the University of Sheffield. *Tg(mpeg1:Gal4.VP-16)sh256* was crossed to *Tg(UAS:Kaede)s1999t* to enable visualisation of macrophages.

### Expression analysis

Zebrafish *IL-1β* (GenBank accession: AY340959.1) was cloned using 20 bp complementary primers (drIL-1βF: 5′-ATGGCATGCGGGCAATATGA-3′, drIL-1βR: 5′-CTAGATGCGCACTTTATCCT-3′) into dual promoter TOPO TA (Life Technologies, UK). Digoxigenin-labelled sense and antisense RNA probes were synthesised (Roche Diagnostics, UK) and WISH performed as previously described (Nüsslein-Volhard and Dahm, 2002). For antibody, TSA and FISH staining, the protocol was adapted from Prajsnar et al. (Prajsnar et al., 2012). Larvae were fixed in 4% w/v paraformaldehyde in PBS overnight at 4°C. Fixed larvae were briefly washed twice in PBS. Larvae were incubated in 1:50 TSA Cyanine5 (Perkin Elmer, UK) without light for 10 minutes at 28°C. Larvae were re-fixed for 20 minutes and endogenous peroxidase activity quenched with a 1-hour incubation in 0.3% H_2_O_2_. Larvae were probed for *IL-1β* mRNA as described in Elworthy et al. (Elworthy et al., 2008) for FISH using anti-digPOD (at 1:10,000) and TSA Cyanine3. The larvae were then stained with an anti-L-plastin antibody as described (Feng et al., 2010) using an Alexa-Fluor-488 anti-rabbit secondary antibody.

### Fixed-sample imaging

Low-resolution images were acquired using a Nikon SMZ1500 stereomicroscope with a DS-Fi1 camera using NIS elements software (Nikon, Japan). Higher-resolution images were acquired on glycerol-mounted embryo tails and imaged on a Bx51 compound microscope, with a Camedia C-3030ZOOM camera using a 40× NA 1.15 water-immersion objective and Cell B software (Olympus, UK). FISH images were acquired on mounted embryo tails using an UltraVIEW VoX spinning disk confocal microscope (Perkin Elmer) on a Axiovert 200M (Zeiss, UK) running Volocity™ software for image acquisition (Perkin Elmer) and using a 40× PlanFLN NA 1.3 oil-immersion objective.

### Agilent microarray expression analysis

Six adult *Tg(mpx:GFP)i114* fish were macerated using a scalpel and passed through a series of graded μm meshes, dissociating the tissue. Cells were sorted by FACS analysis into cells showing neutrophil-like side scatter (SSC) and forward scatter (FSC), and further sorted into GFP-positive and -negative (control) sets. RNA was extracted using the mirVana™ kit (Life Technologies). Microarray analysis was performed using a Zebrafish (V3) Gene Expression Microarray containing 43663 probes (Agilent Technologies, The Netherlands).

### 3D-structure prediction

The 3D model of zebrafish IL-1β was generated by submitting the protein sequence (accession: NP_998009.1) to the Phyre Server (Kelley and Sternberg, 2009).

### Treatment with IL-1β-pathway inhibitors

Embryos were pre-treated with compounds as described, for 2 hours before injury. Embryos were injured by tail transection at the distal-most point of the pigment gap at 3 dpf as described (Renshaw et al., 2006) and neutrophil or macrophage numbers at the site of injury counted at 6 hpi. *Tg(pNF-κB:EGFP)sh235* embryos were injured as described, mounted in 0.75% LMP agarose at 8 hpi and imaged with a 10× NA 0.3 objective using a TE-2000U microscope (Nikon) and an Orca-AG camera (Hamamatsu, Japan) using Volocity™. The mean fluorescent intensity was measured between the line of injury and proximal end of the pigment gap, ignoring neuromasts, using Volocity™ for quantification.

### Analysis of gene expression

Leukocyte-specific RNA was obtained by dissociating *mpeg:gal4;UAS:Kaede* or *mpx:GFP* embryos in PBS with 0.4% trypsin on an orbital shaker for 1 hour with frequent trituration using a pipette. Cells were sorted by FACS analysis and RNA extracted using an RNA Isolate II micro kit (Bioline, UK). Tail-tissue RNA was obtained as described (de Oliveira et al., 2013) using Superscript 2 (Invitrogen). qRT-PCR was performed on a CFX96 detection system (Bio-Rad, UK) using Dynamo Flash SYBR green mastermix (Thermo Scientific, UK) using primers described in de Oliveira et al. (de Oliveira et al., 2013).

### Statistical analysis

Data were analysed using Prism 6 (Graphpad, USA). Data shown are mean ± s.e.m. using multiple *t*-tests with a Bonferroni correction or one-way ANOVA with Dunnet’s post-test.
